# Versatile roles of inositol hexakisphosphate in the ubiquitin-proteasome system

**DOI:** 10.1042/EBC20253032

**Published:** 2026-02-13

**Authors:** Domnita-Valeria Rusnac, Ning Zheng, Xiaowen Xie

**Affiliations:** 1 Department of Pharmacology, University of Washington, Seattle, WA, 98195, U.S.A.; 2 Howard Hughes Medical Institute, University of Washington, Seattle, WA, 98195, U.S.A.; 3 School of Pharmaceutical Sciences, Peking University, Beijing, 100191, China

**Keywords:** allosteric modulator, inositol hexakisphophate (IP6), molecular glue, structural cofactor, ubiquitin-proteasome system (UPS)

## Abstract

Inositol hexakisphosphate (IP6) is an endogenous organic molecule present in eukaryotes. First characterized as a phosphorus-storage metabolite, it has been subsequently discovered to play a role in modulating a multitude of different biological pathways. Here, we provide a concise overview of the involvement of IP6 in the ubiquitin-proteasome system (UPS). As an allosteric regulator, a molecular glue, and a potential prosthetic group, IP6 directly impacts the activities of multiple major UPS components. We specifically highlight the structural mechanisms through which IP6 binds to individual proteins or multi-protein complexes to control their functions. The serendipitous discovery of IP6 in various protein structures raises questions about the prevalence, identity, and regulation of soluble inositol polyphosphates in the UPS, which have potential translational implications.

## Introduction

**Figure 1:**
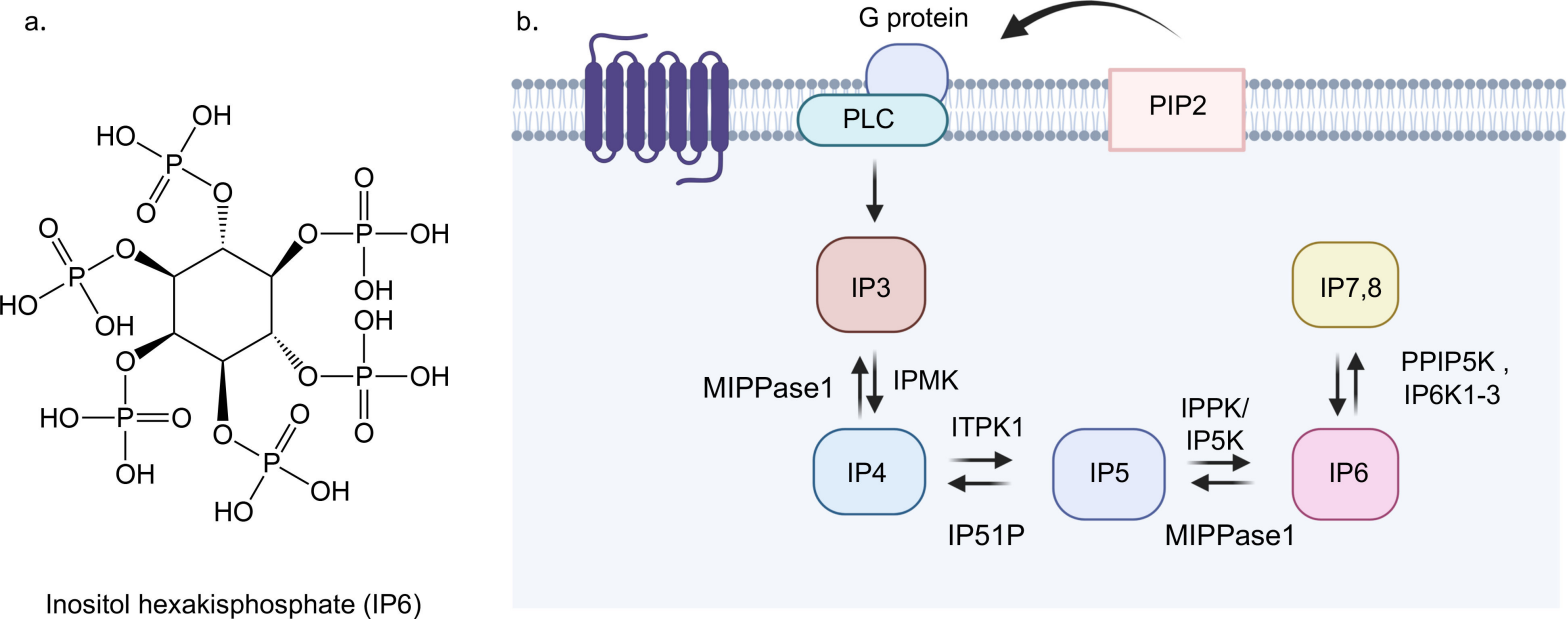
IP6 biosynthetic pathway.

Inositol hexakisphosphate (IP6) is one of the most abundant inositol polyphosphates in eukaryotic cells. Its synthesis begins with myo-inositol and proceeds through a sequential phosphorylation cascade (Figure 1a). This process is initiated by the hydrolysis of phosphatidylinositol 4,5-bisphosphate by phospholipase C to generate inositol 1,4,5-trisphosphate (IP3), which is a well-known second messenger [1–4]. Subsequent phosphorylation of IP3 mediated by a series of kinases, including inositol polyphosphate multikinase and inositol tetrakisphosphate 1-kinase, produces inositol pentakisphosphate (IP5) [5]. The final step of IP6 synthesis is catalyzed by inositol pentakisphosphate 2-kinase (IPPK/IP5K), which phosphorylates IP5 at the 2-position to yield fully phosphorylated IP6 [6,7]. Beyond its role as a biosynthetic end product, IP6 serves as a precursor for inositol pyrophosphates, for example, IP7 and IP8. These derivatives, produced by IP6 kinases (IP6K1–3) and PPIP5Ks, have been implicated in energy sensing, apoptosis regulation, and phosphate homeostasis [8,9] (Figure 1b).

a. IP6 structure. b. IP6 synthesis is involved with multiple enzymes. Gαq, Gq protein α subunit; IP3, inositol trisphosphate; IP4, inositol tetrakisphosphate; IP5, inositol pentakisphosphate; IP51P, IP5 phosphatase 1; IP6, inositol hexakisphosphate; IP7,8, inositol pyrophosphates; IPMK, inositol polyphosphate multikinase; IPPK/IP5K, inositol pentakisphosphate 2-kinase; IP5K1–3, IP6 kinases; ITPK1, inositol tetrakisphosphate 1-kinase; MIPPase1, multiple inositol polyphosphate phosphatase; PIP2, phosphatidylinositol 4,5-bisphosphate; PLC, phospholipase C, PPIP5K, diphosphoinositol pentakisphosphate kinase.

Traditionally viewed as a signaling metabolite or phosphate-storage molecule, IP6 is now recognized as a versatile and evolutionarily conserved molecular cofactor. Emerging structural and biochemical studies reveal that IP6 contributes to diverse biological processes by providing architectural stability, instead of acting as a transient signal. It binds with high specificity and durability to protein complexes, stabilizing multi-subunit assemblies and altering enzymatic activity. Noticeably, IP6 was mostly identified by fortuity from structural studies. In the majority of, if not all cases, IP6 was unexpectedly found from a non-proteinaceous density associated with a protein of interest that is not related to IP6 biosynthesis. These include functionally diverse proteins, such as RNA-editing enzymes [10], transcriptional regulators [11,12], and mRNA-exporting regulators [13,14], as well as protein complexes involving ubiquitin E3 ligases [15–17]. In these cases, IP6 binds to positively charged pockets via electrostatic interactions in which the interacting residues are well conserved. These findings elevate IP6 to the status of general-purpose cofactors such as ATP, SAM, and NAD^+^, which serve not merely as energy carriers or redox agents but also as fundamental regulators of protein architecture and function [18–22].

One area where IP6’s regulatory role is increasingly evident is the ubiquitin-proteasome systems (UPSs), the primary pathway for targeted protein degradation in eukaryotic cells.

**Figure 2:**
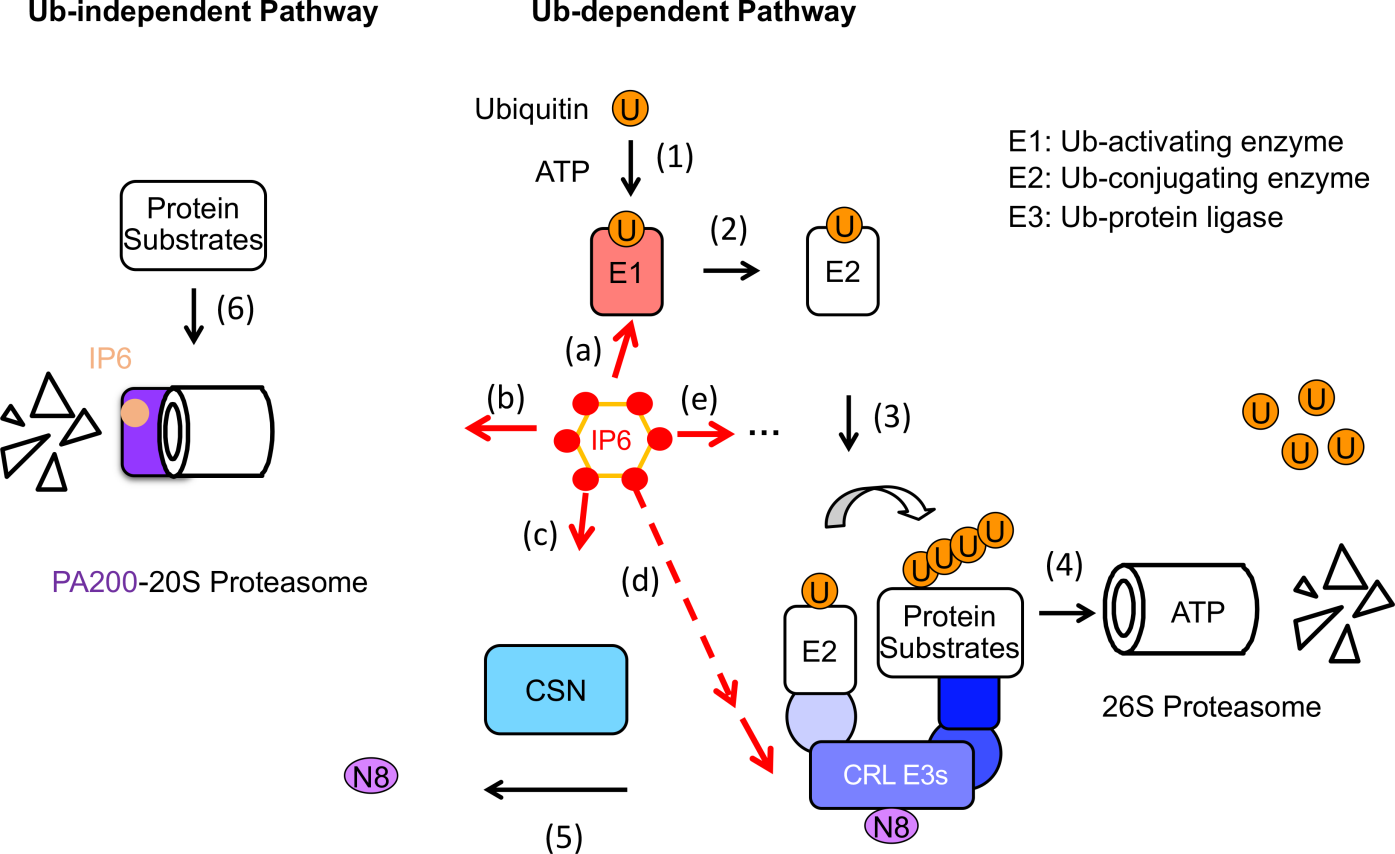
IP6 plays multiple roles in the ubiquitin-proteasome system.

The UPS governs critical cellular processes such as cell cycle progression, signal transduction, and stress responses [23,24]. Protein degradation begins with the covalent attachment of ubiquitin to substrates via an enzymatic cascade involving an E1 ubiquitin-activating enzyme, an E2 ubiquitin-conjugating enzyme, and an E3 ubiquitin ligase, which confers substrate specificity (Figure 2). Cullin-RING ligases (CRLs) constitute the largest family of E3s and require activation through a post-translational modification known as neddylation—the covalent attachment of the ubiquitin-like (Ubl) protein Neural precursor cell expressed developmentally down-regulated protein 8 (NEDD8) to the cullin scaffold [25,26]. Neddylation has been shown to trigger conformational changes of cullin scaffold, which promote ubiquitin transfer [27]. This modification is reversed by the COP9 signalosome (CSN), which removes NEDD8 [28,29]. However, by facilitating the exchange of substrate receptor subunits of CRLs, CSN also plays a positive role in regulating CRL E3s. Polyubiquitinated proteins are ultimately recognized and degraded by the 26S proteasome, a multi-subunit protease complex that ensures timely and selective protein turnover [30,31].

UPS consists of ubiquitin-dependent and ubiquitin-independent pathways, both of which involve IP6 as a regulatory molecule. Black arrows indicate different steps of ubiquitin-dependent degradation pathway and ubiquitin-independent pathway, including (1) activation by E1; (2) ubiquitin transfer to E2; (3) substrate polyubiquitination catalyzed by E3; (4) recognition and degradation of polyubiquitinated substrate protein by the 26S proteasome; (5) CRL deneddylation by CSN. N8 labels NEDD8; (6) substrate degradation by PA200-20S proteasome. Red arrows indicate the different targets of IP6 and its functional roles. These include IP6: (**a**) as an allosteric regulator of UBA6 E1; (**b**) as a potential prosthetic group of PA200; (**c**) as a molecular glue acting on CSN and CRL; (**d**) as a molecular glue acting on E3s and substrates; (**e**) as a potential interactor of other proteins of the UPS. Ub or U: ubiquitin.

In this review, we highlight the emerging roles of IP6 in regulating the UPS at multiple levels (Figure 2): (1) as an allosteric regulator modulating conformational transitions of the E1 enzyme UBA6 (Ubiquitin Like Modifier Activating Enzyme 6 ); (2) as a molecular glue bridging LSD1–HDAC–CoREST (Lysine-specific histone demethylase 1-Histone Deacetylase-REST corepressor 1) substrate complex to CRL3^KBTBD4^ E3 ligase or stabilizing CRL–CSN assemblies; (3) as a potential prosthetic group embedded within key UPS components, such as the auxin receptor E3 ligase TIR1 (Protein Transport Inhibotor Response 1) and the PA200 proteasome activator; and (4) as a potential modulator of yet-to-be-characterized IP6-binding UPS factors. By highlighting IP6 as an underappreciated regulator of proteostasis, we discuss the current understanding, emerging hypotheses, and future opportunities for harnessing IP6 in the therapeutic modulation of the UPS.

### IP6 as an allosteric regulator of UBA6 E1

The E1 ubiquitin-activating enzyme activates ubiquitin by using ATP-Mg^2+^ to catalyze the acyl adenylation of ubiquitin’s C-terminal carboxyl group. This is followed by the formation of a thioester bond between the activated ubiquitin and the catalytic cysteine residue of the E1. In vertebrates and sea urchins, two genes encode the E1 enzymes, UBE1 (Ubiquitin-activating enzyme E1) and UBA6, which preferentially charge distinct subsets of E2 enzymes [32]. UBA6 differs from UBA1 by activating not only ubiquitin but also the Ubl modifier FAT10 (Human leukocyte antigen (HLA)-F adjacent transcript 10) [33,34], which has been implicated in a range of biological processes, such as polyglutamine diseases [35], mitotic progression [36], immunity [37–40], and cancer development [41–44].

**Figure 3:**
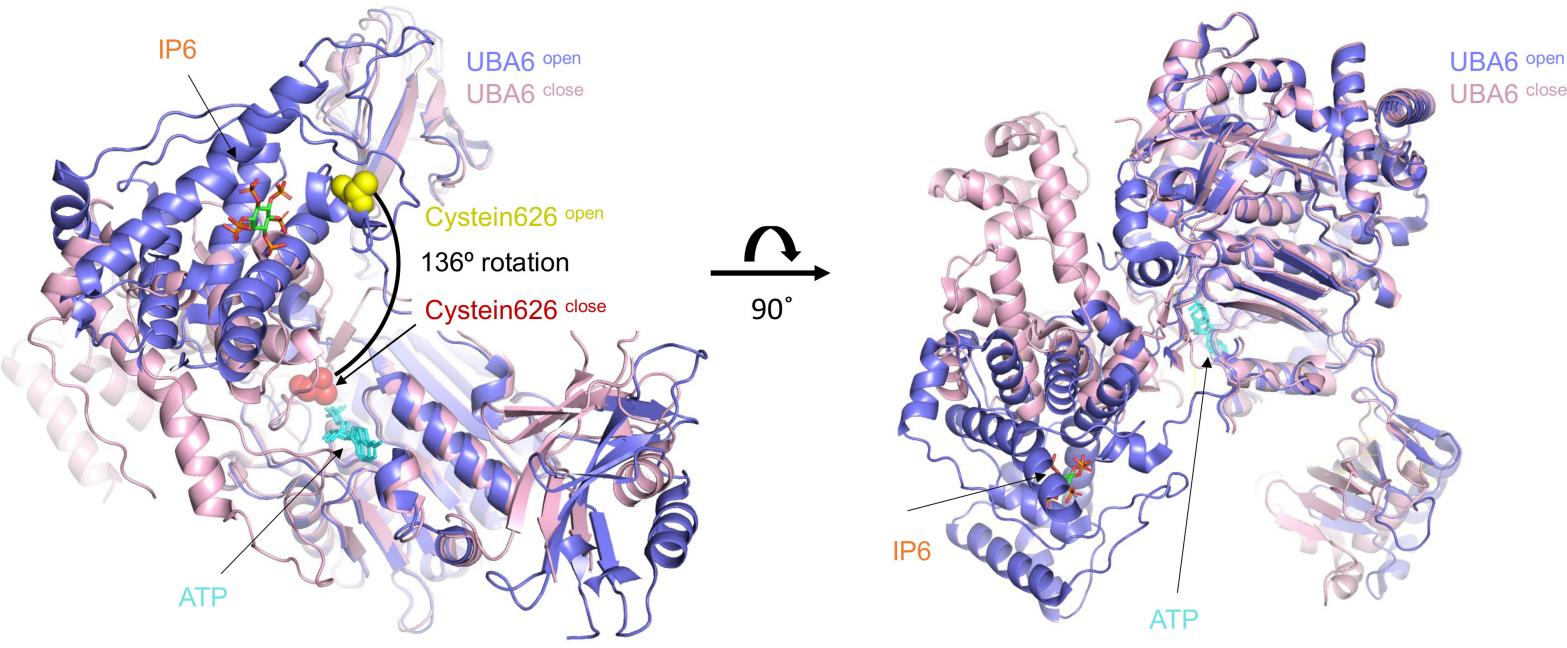
Superposition of UBA6 open and UBA6 close shows the binding of IP6 inhibiting the conformational rearrangement required for the E1~ubiquitin thioester bond formation.

In 2022, multiple groups set out to better understand how UBA6 catalyzes the activation of ubiquitin and FAT10 and characterized UBA6 in the context of multiple complexes. One study by Lingmin Yuan et al. revealed that the crystal structure of the human UBA6–ubiquitin complex displayed two markedly different conformations: an open form set for adenylation and a closed form primed for thioester bond formation [45]. Notably, in the open conformation, an IP6 molecule was found bound to a highly basic, evolutionarily conserved pocket unique to UBA6. By interacting with multiple residues and structural motifs, IP6 inhibits the conformational rearrangement required for thioester bond formation. Consistent with this proposed mechanism, IP6 was absent from the closed conformation (Figure 3). Structural and biophysical analyses demonstrated that IP6 acts as an allosteric inhibitor of UBA6 by modulating the conformational switch between the open and closed states, while simultaneously enhancing the structural stability of the enzyme. These findings not only elucidate the structural basis of UBA6’s enzymatic activity but also reveal a novel mode of regulation by a naturally occurring metabolite. The high affinity between UBA6 and IP6 (42 nM) and the high intracellular levels (tens of μM) of IP6 suggest that the E1 might be constantly inhibited until IP6 is freely released by an unknown mechanism [46]. Notably, how much IP6 is freely available remains unclear, and thus these IP6-binding interfaces could also be potentially regulated by free IP6 fluctuations. Of note, this discovery was made possible only because IP6 was serendipitously co-purified with UBA6 from insect cells. In contrast, Ngoc Truongvan et al. purified and determined the structure of the E1 enzyme expressed in E. *coli*, which does not produce IP6, and did not observe its presence in the structures [47].

UBA6 open, shown in blue cartoon, is super positioned with UBA6 close, shown in pink (PDB ID: 7SOL); IP6, bound to an allosteric pocket on UBA6, is shown in sticks with orange colored phosphate groups and green-colored cyclohexane ring; ATP is shown in cyan sticks; Cys626 open (in yellow spheres) is related to its position in the closed state, C626 close (in red spheres) by a 136° rotation.

### IP6 as a molecular glue

Molecular glue is a unique class of proximity inducers distinct from bifunctional molecules that require a high binding affinity to both protein targets [48–50]. First identified in the UPS, molecular glues are monovalent small molecules that can promote protein–protein interactions by complementing their suboptimal interfaces. In the UPS, these small molecules have been shown to enable ubiquitin ligases to target natural or neo-substrates for proteasomal degradation without showing a detectable affinity to the substrate proteins [49,50]. Due to their excellent pharmacokinetic properties and unique mechanisms of action, molecular glue degraders allow unprecedented therapeutic targeting of proteins that are conventionally deemed undruggable [51–54]. At the end of the E1-E2-E3 cascade, E3 ubiquitin ligases function by simultaneously engaging a ubiquitin-charged E2 enzyme and a specific substrate. IP6 has been found to directly modulate substrate recognition and complex assembly by E3 ligases as a molecular glue.

### IP6 as a molecular glue of an E3 ligase and its neo-substrate

**Figure 4:**
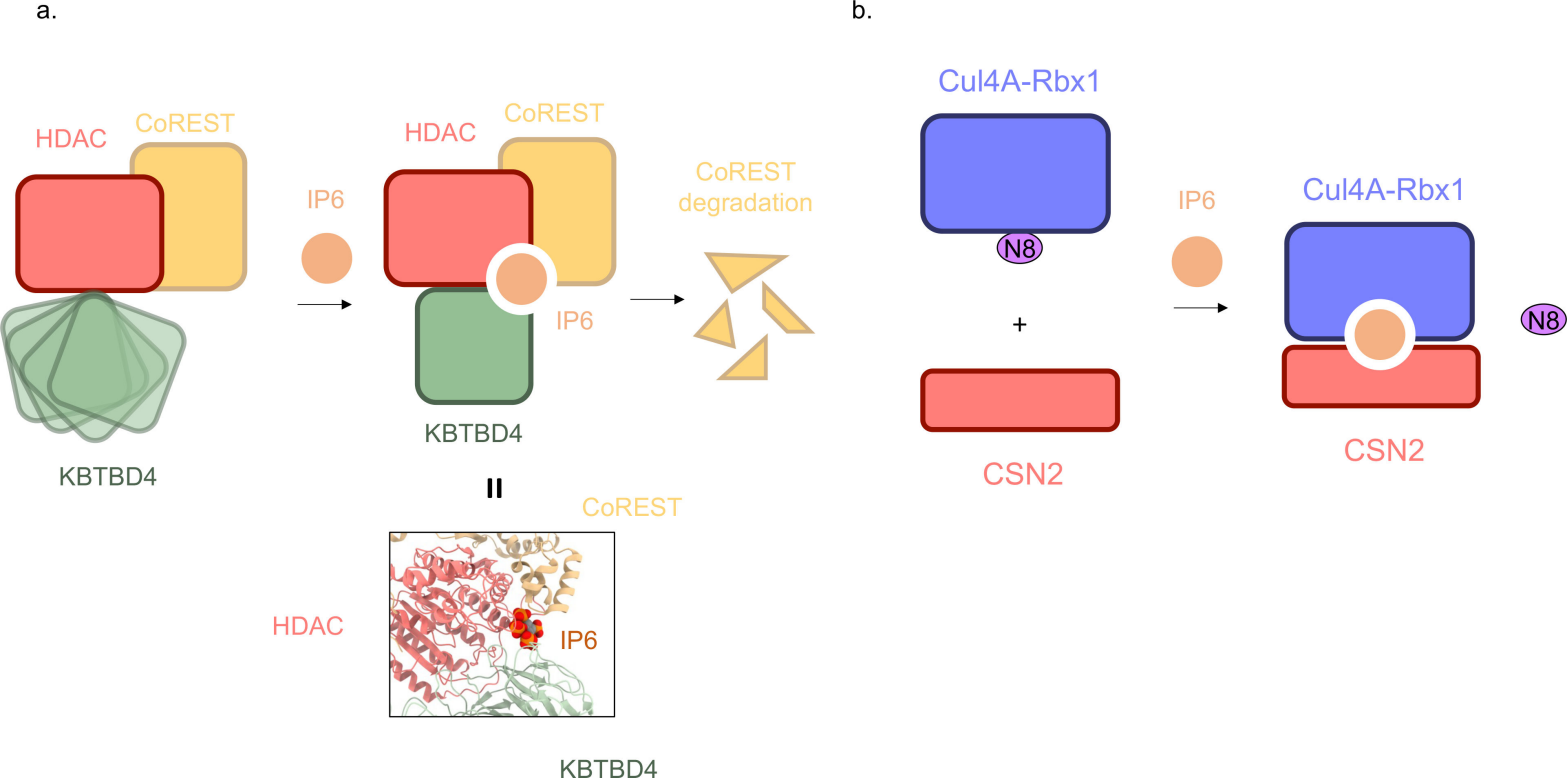
IP6 functions as a molecular glue acting on E3 ligases and their regulators.

Recent structural and biochemical studies have revealed that IP6 acts as a molecular glue facilitating neo-substrate recognition by the CRL3^KBTBD4^ E3 ubiquitin ligase complex. As a substrate receptor of CRL3, KBTBD4 (Kelch repeat and BTB domain-containing protein 4) acquires neomorphic substrate-binding activity through cancer-associated indel mutations identified in medulloblastoma [55]. It has been shown that these mutations confer gain-of-function to the E3 to promote the degradation of select subunits in the HDAC1/2-containing transcriptional co-repressor complexes, including CoREST and LSD1 [15,56]. Xie et al. determined high-resolution cryo-EM structures of KBTBD4 carrying medulloblastoma-associated insertions (R313delinsPRR and IPR310delinsTTYML mutants) bound to LSD1–HDAC1–CoREST [15]. These structures revealed an IP6 molecule at the trimolecular junction of HDAC1, CoREST, and KBTBD4. Specifically, IP6 makes direct contact with all three proteins, contributing to the stabilization of the cancer mutation-enabled neo-substrate-E3 complex (Figure 4a). A parallel study by Yeo et al. reported a similar role for IP6 in the wildtype KBTBD4 bound to LSD1–HDAC1–CoREST, an assembly that is glued together by a small-molecule degrader known as UM171 [16]. In this structure, IP6 is situated similarly at the three-molecule junction of HDAC1, CoREST, and KBTBD4, helping further cement the complex as a second molecular glue. Both studies show that IP6 dramatically enhances substrate binding by ~25 fold in a TR-FRET (Time-Resolved Fluorescence Resonance Energy Transfer ) assay. Importantly, IP6 is not just a structural cofactor of the HDAC1-CoREST complex as previously characterized [57]. In the E3-neo-substrate assembly, it is essential for the neomorphic substrate interaction, as disruption of IP6 co-ordination by KBTBD4 abolished neo-substrate recruitment and downstream ubiquitination. These findings illustrate how metabolic signaling molecules like IP6 can help reshape substrate specificity of E3 ligase complexes with implications for targeted degradation therapeutics.

a. IP6 stabilizes the complex formation between KBTBD4 (green rectangle), the substrate receptor of a CRL3 complex, and HDAC (salmon rectangle)/CoREST (yellow rectangle), promoting uibiquitin transfer and subsequent degradation of the neosubstrate CoREST; IP6 (red/orange/grey spheres) achieves this effect by simultaneously interacting with HDAC, CoREST and KBTBD4, shown in salmon, yellow, and green cartoon, respectively. For clarity, the rest of the CRL3 complex is omitted. IP6 in red/orange/grey spheres; b. IP6 bridges CSN2 with Cul4A to facilitate the removal of NEDD8 (Purple oval labeled N8) from Cul4A.

### IP6 as a molecular glue of CSN and CRLs

In addition to supporting substrate recruitment, IP6 also regulates CRLs by stabilizing their interactions with CSN (Figures 2 and 4b). Scherer et al. showed that IP6 mediates an electrostatic bridge between the acidic N-terminal tail of CSN subunit 2 (CSN2) and a conserved basic canyon on cullins, enhancing the CSN–CRL affinity by ~30 fold with an EC₅₀ of about 20  nM [58]. Lin et al. confirmed this structurally by determining a cocrystal structure of IP6 with CSN2, which revealed several conserved lysine residues on CSN2 and the RING protein Rbx1 responsible for co-ordinating IP6. This binding mode of IP6 is consistent with a corresponding unassigned cryo-EM density at the CSN–CRL interface [59]. In this configuration, IP6 blocks the E2 enzyme (CDC34) from docking to the cullin-RING scaffold and promotes CSN5-catalyzed removal of NEDD8. In corroboration with this mechanism, an IP6-binding-deficient CSN2 mutant (Lys70→Glu) fails to recruit CSN to CRLs and is embryonic lethal. Functionally, IP6-driven CSN binding inactivates CRLs and protects proteostasis. For example, depletion of IP5K, the kinase that generates IP6, leads to cullin hyper-neddylation and accelerated degradation of CRL substrates, such as p27 and p21 [58]. Notably, the IP6 ‘molecular glue’ is dynamically regulated by inositol phosphate metabolism. IP6K1 converts IP6 to IP7, which weakens CSN–CRL interactions and favors complex dissociation [60]. This dynamic regulation links inositol phosphate metabolism to proteostasis, positioning IP6 as a molecular switch that co-ordinates metabolic state with ubiquitin ligase activity.

## IP6 as a potential prosthetic group

### IP6 as a potential prosthetic group of an E3 ligase

When elucidating the mechanism by which the TIR1 E3 ubiquitin ligase perceives auxin, a pivotal plant hormone that controls plant growth and development, Tan et al. were the first to discover IP6 in a UPS complex [50]. IP6 was revealed in the structure as a tightly bound cofactor of TIR1 upon its fortuitous copurification with the TIR1–ASK1 (*Arabidopsis* skp1-like 1) complex from insect cells. The identity of the metabolite was further confirmed by native protein mass spectrometry analysis. Importantly, the majority of TIR1 residues that interact with IP6 are positively charged and strictly conserved among the TIR1 paralogues and orthologues in plants, suggesting that IP6 binding is essential for this specific subfamily of proteins. Considering auxin is a growth hormone in plants, it is plausible that IP6 might serve as a proxy signal for phosphorus abundance, which is a prerequisite for auxin perception and signaling by TIR1.

### IP6 as a potential prosthetic group of proteasome

With the modification by Lys-48-linked polyubiquitin chain [61], UPS substrates are recognized and degraded by the 26S proteasomes in a highly regulated, ubiquitin- and ATP-dependent manner. While many 20S catalytic core particles are coupled to the 19S regulatory particles to form 26S proteasome particles, a subset of 20S core particles can complex with the PA200 activators, giving rise to ATP- and ubiquitin-independent proteasomes (Figure 2). These alternative proteasomes are present in most mammalian tissues at low levels, except in the testes, where 90% of the proteasomes contain at least one PA200 subunit. The PA200-20S proteasome has been implicated in DNA repair, chromatin remodeling, spermatogenesis, and the degradation of acetylated histones [62–66].

Recent cryo-EM structures of the human PA200-20S complex, determined by Rêgo et al. and Guan et al*.,* shed light on the activation mechanism of these atypical proteasomes [67,68]. PA200 binding induced structural rearrangements in the α-ring of the 20S catalytic core, resulting in partial opening. Moreover, the PA200 structure revealed two openings lined with positively charged residues, which are believed to serve as the entry sites for unfolded proteins. Unexpectedly, both groups observed clear non-proteinaceous densities occupying these PA200 sites, which they later assigned to IP6 and 5,6[PP]2-IP4. The authors speculate that the inositol polyphosphates could affect the degradation of acetylated histones due to the preexisting connection between HDACs and IPs. While the inositol polyphosphates do not seem to completely obstruct the entryway to the catalytic core, they alter the passageway serving as a potential size or charge filter. It remains unclear if IP6 and 5,6[PP]2-IP4 are permanent cofactors for PA200 and how their binding to the proteasomes is regulated.

### Other potential IP6 interacting proteins in the UPS

While the structural roles of IP6 in multiple cases are now well-defined, additional evidence suggests that IP6 may influence a broader range of the UPS components. In cases where direct structural insights are still lacking, biochemical and genetic studies point to a regulatory function for IP6 in other E3 ligases and their substrate interactions. One such example comes from plant biology, where IP6 appears to modulate substrate recognition in a RING-type E3 ligase involved in nutrient homeostasis. Nitrogen limitation adaptation protein (NLA) is a plant RING E3 ubiquitin ligase that regulates phosphate homeostasis. It targets the phosphate-starvation response master transcription factor PHR1 (Phosphate Starvation Response 1) for ubiquitination and degradation, thereby attenuating phosphate uptake genes when phosphate is sufficient [69]. Park et al. showed that NLA’s interaction with PHR1 is modulated by inositol polyphosphates. It has been demonstrated *in vitro* that IP6, but not IP5, dramatically enhances NLA’s binding to PHR1 and promotes PHR1 polyubiquitination. Mutational or genetic evidence further supports this conclusion. *Arabidopsis thaliana* mutants with altered IP6/IP7 levels showed corresponding changes in PHR1 stability: high PHR1 accumulation in an IPK1/ITPK mutant that lowered IP7, which is consistent with reduced NLA activity. Bioinformatic analyses have noted that the SPX domain (a domain is found at the amino terminus of a variety of proteins, including SYG1, Pho81 and XPR1) of NLA (Arabidopsis nitrogen limitation adaptation protein) carries a conserved basic surface known in other SPX proteins to bind IP6/IP7. Indeed, crystal structures of unrelated proteins’ SPX domains of yeast Vtc have captured IP6 bound in a basic cleft, supporting the predicted IP6-binding mode in NLA [70].

## Conclusions

IP6, which has long been considered a metabolic byproduct or phosphate-storage molecule, is now emerging as a structurally and functionally essential cofactor in the UPS and beyond. As the regulation of protein degradation affects nearly every aspect of cellular homeostasis and disease, understanding the non-canonical modulators of this system, including metabolites like IP6, is of growing importance. With six phosphate groups, IP6 preferentially engages clusters of positively charged residues binding sites that may reside within a single polypeptide or at protein–protein interfaces. Although the interacting basic residues are often well conserved, they are usually not associated with a dedicated domain with a particular fold. Therefore, the IP6-binding pocket can be accommodated by very diverse protein structures. Similar features of IP6-binding sites have also been observed outside the UPS [10,12,14]. Such a structural and sequence promiscuity explains why IP6 can assume multiple functional roles depending on the biological context. These include, but are not limited to, stabilizing protein–protein interfaces, altering local structure, modulating global conformational states, and supporting active sites. Through this review, we highlighted the versatile roles of IP6 in regulating protein homeostasis as an allosteric regulator, a molecular glue, and a potential prosthetic group across diverse components of the UPS. Although the studies we discussed in this article showcase the multifaceted roles of IP6 in the UPS, many questions remain.

First of all, is IP6 the bona fide modulator of the proteins of interest? Given its high negative charge density, IP6 has an intrinsic tendency to bind to positively charged protein surfaces with concentrated positive charges. While indicative of a functional role, co-purification of IP6 with a protein of interest is not sufficient to fully establish its biological relevance. Second, in the context of the proteins of interest, is IP6 the physiologically relevant form of inositol polyphosphates? Emerging evidence has suggested that other inositol polyphosphates (e.g. IP7) and enzymes converting IP6 to these inositol polyphosphates may also be active players, if not the primary regulators, in certain pathways [58,59,71].

Third, assuming IP6 is indeed the bona fide modulator of the target proteins, is its level regulated in different cellular contexts presumably by enzymes such as IP6K1-3 and multiple inositol polyphosphate phosphatase? Losing or gaining a phosphate group could alter the ability of IP6 to engage its targets and elicit cascading effects [59]. As a result, IP6 metabolites may promote a more dynamic protein assembly than what IP6 alone can achieve [71]. Therefore, modulating the dynamic balance between IP6 and its metabolites could be an important intervention approach to regulate the UPS.

The UPS represents an emerging and exciting target of therapeutic manipulation. In fact, E1 [72,73], E2 [74,75], E3 [76,77], CSN [78,79], and proteasome [80,81] have all been targeted pharmacologically. Given the close involvement of IP6 in the UPS, it is conceivable that the enzymes responsible for maintaining the cellular level of IP6 could also be regulated with small molecules for therapeutic benefits. The dual nature of IP6 as both a dynamically regulated metabolite and an architectural modulator positions it as a promising regulatory node for interventions in cancer, neurodegeneration, and other proteostasis-related diseases. In fact, IP6-binding deficient mice can avoid hyperinsulinemia stress and its role in safeguarding insulin secretion in beta cells [82]. Together, these perspectives highlight a paradigm shift in how we understand and manipulate the UPS.

**Summary points**

- Inositol hexakisphosphate (IP6) serves as an allosteric regulator of enzymatic conformational changes, exemplified by its inhibition of UBA6-mediated ubiquitin and FAT10 activation.

- IP6 functions as a molecular glue in CRL3 and COP9 signalosome (CSN complexes, bridging not only substrate adaptor KBTBD4 and neo-substrate LSD1–HDAC–CoREST but also CRL E3s and CSN to regulate protein ubiquitination.

- IP6 acts as a potential prosthetic group that binds proteasome activator PA200 and supports the E3 ligase activity of TIR1.

- IP6 acts as a key multifaceted and versatile metabolite that modulates protein functionality across multiple levels of UPS.

- Future efforts to unravel new IP6-binding proteins, decode inositol-phosphate signaling hierarchies, and modulate IP6 dynamics could open new avenues for targeting proteostasis in disease.

## Open Access

This article has been published open access under our Subscribe to Open programme, made possible through the support of our subscribing institutions, learn more here: https://portlandpress.com/pages/open_access_options_and_prices#conditional

## Abbreviations

CRL: Cullin-RING E3 ligase
CSN: COP9 signalosome
CSN2: CSN subunit 2
IP3: inositol 1,4,5-trisphosphate
IP5: inositol pentakisphosphate
IP6: inositol hexakisphosphate
IPPK: inositol pentakisphosphate 2-kinase
NLA: nitrogen limitation adaptation protein
UPS: ubiquitin-proteasome system
Ubl: ubiquitin-like
